# Mitochondrial interaction of fibrosis-protective 5-methoxy tryptophan enhances collagen uptake by macrophages

**DOI:** 10.1016/j.freeradbiomed.2022.06.235

**Published:** 2022-06-23

**Authors:** Sjors Maassen, Harry Warner, Melina Ioannidis, Jack Jansma, Hugo Markus, Sahar El Aidy, María-Dolores Chiara, Jose Luis Chiara, Larissa Maierhofer, Helen Weavers, Geert van den Bogaart

**Affiliations:** a Department of Molecular Immunology, Groningen Biomolecular Sciences and Biotechnology Institute, University of Groningen, Groningen, 050, the Netherlands; b Department of Host-Microbe Interactions, Groningen Biomolecular Sciences and Biotechnology Institute, University of Groningen, Groningen, 050, the Netherlands; c Institute of Sanitary Research of the Principado de Asturias, Oviedo, Spain; d CIBERONC (Network of Biomedical Research in Cancer), Madrid, Spain; e Institute of General Organic Chemistry, IQOG-CSIC, Madrid, Spain; f School of Biochemistry, Biomedical Sciences, University of Bristol, Bristol, BS8 1TD, United Kingdom; g Department of Medical Biology and Pathology, University Medical Center Groningen, University of Groningen, 050, the Netherlands

**Keywords:** Macrophage, IDO, Mitochondria, Metabolism, Inflammation, Fibrosis, 5-Methoxy tryptophan

## Abstract

5-methoxy tryptophan (5-MTP) is an anti-fibrotic metabolite made by fibroblasts and epithelial cells, present in a micromolar concentrations in human blood, and is associated with the progression of fibrotic kidney disease, but the mechanism is unclear. Here, we show by microscopy and functional assays that 5-MTP influences mitochondria in human peripheral blood monocyte-derived macrophages. As a result, the mitochondrial membranes are more rigid, more branched, and are protected against oxidation. The macrophages also change their metabolism by reducing mitochondrial import of acyl-carnitines, intermediates of fatty acid metabolism, driving glucose import. Moreover, 5-MTP increases the endocytosis of collagen by macrophages, and experiments with inhibition of glucose uptake showed that this is a direct result of their altered metabolism. However, 5-MTP does not affect the macrophages following pathogenic stimulation, due to 5-MTP degradation by induced expression of indole-amine oxygenase-1 (IDO-1). Thus, 5-MTP is a fibrosis-protective metabolite that, in absence of pathogenic stimulation, promotes collagen uptake by anti-inflammatory macrophages by altering the physicochemical properties of their mitochondrial membranes.

## 1 Introduction

Macrophages are plastic immune cells that adapt to their environment and play critical roles in all stages of disease: inflammation, resolution, and tissue repair [1]. Macrophages can be either tissue-resident or differentiated from monocytes that traverse into tissues from the blood. During the differentiation of macrophages, environmental signals in the tissues shape their phenotype. Microbial components (*e.g*., lipopolysaccharide (LPS)) and pro-inflammatory cytokines (*e.g*., tumour necrosis factor-alpha (TNF-α)) polarize macrophages towards an inflammatory phenotype, often referred to as classically-activated or M1-like macrophages. These inflammatory macrophages are associated with the clearance of pathogens and inflammatory signaling to other cells. In contrast, anti-inflammatory cytokines (*e.g*., Interleukin-4) result in differentiation of anti-inflammatory macrophage phenotypes, also referred to as alternatively-activated or M2-like macrophages. These anti-inflammatory macrophages play roles in scavenging of extracellular matrix (ECM) and tissue homeostasis. However, macrophage differentiation is complex, with many intermediate phenotypes being described with unique and shared phenotypes [2], and the variety of stimuli that drive macrophage differentiation are incompletely understood.

The function of macrophages coincides with, and is regulated by, alterations in metabolic activity referred to as the immunometabolism [3]. To fulfil their effector functions, such as phagocytosis, killing of pathogens, and cytokine production, M1 macrophages have increased glycolysis, elevated pentose phosphate pathway and fatty acid synthesis, and a truncated Krebs cycle leading to the accumulation of citrate, succinate and itaconate [4,5]. Pro-inflammatory macrophages also uncouple their electron transport chain, thereby promoting the formation of reactive oxygen species (ROS) [6]. Higher ROS levels promote lipid oxidation and formation of aldehyde products like 4-hydroxynonenal (4-HNE), which in turn stimulate the fission of mitochondria [7]. As a consequence, pro-inflammatory macrophages generally contain large numbers of fragmented mitochondria. This mitochondrial fission and ROS production aid the clearance of pathogens by macrophages [8,9].

In contrast, M2 macrophages rely more on oxidative phosphorylation and have a lower flux through the pentose phosphate pathway. Anti-inflammatory macrophages thus maintain a more intact respiratory chain and have a lower production of ROS. Hence, anti-inflammatory macrophages display a higher degree of mitochondrial fusion, and their mitochondria usually consist of a large branched network-like structure [10]. Anti-inflammatory macrophages play critical roles in wound healing, as they promote the production of ECM by fibroblasts early during the wound healing, while later they remove excess ECM using scavenger receptors [11]. Insufficient clearance of excess ECM leads to fibrosis. However, the signals that shift macrophages to the removal of excess ECM are still elusive [12].

One candidate macrophage differentiation factor is 5-methoxytryptophan (5-MTP). 5-MTP is a metabolite present in micromolar concentrations in human serum and is made by fibroblasts and epithelial cells [13,14]. Multiple metabolomic studies in human patients found that circulating 5-MTP levels inversely correlate with the disease progression of fibrotic chronic kidney disease [15,16]. Furthermore, 5-MTP exerts numerous antifibrotic effects, for example inhibiting activation of mouse macrophages [17] and blocking fibroblast and hepatic stellate cell differentiation to myofibroblasts [18], and has recently been suggested as a key lead compound for developing new anti-fibrotic drugs [19]. Although 5-MTP has been shown to induce nuclear-erythroid factor 2 (NRF2) signaling [15] and to inhibit P300 histone acetyl transferase by reducing P38 activation and signaling in mouse macrophages [17], it is unknown how 5-MTP affects macrophage differentiation.

## 2 Results

### 2.1 5-MTP increases collagen uptake, but does not affect LPS-induced production of TNF-α

**Fig. 1:**
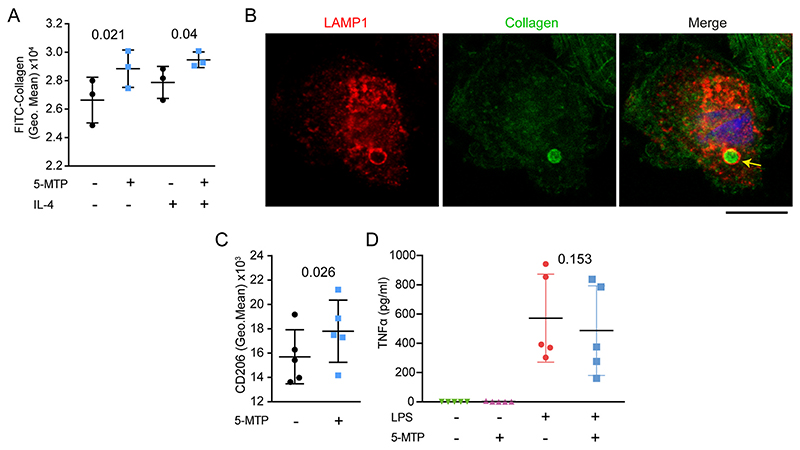
Increased collagen uptake in 5-MTP differentiated macrophages. **A)** Uptake of collagen-FITC by macrophages differentiated with and without 5-MTP after 24 h by flow cytometry with and without IL-4 (*n* = 3 donors, two-way ANOVA with a Sidak’s multiple comparisons test). **B)** Confocal microscopy of 5-MTP differentiated macrophages incubated with FITC-labelled collagen (green). Red: immunostaining for lysosomal marker LAMP1. Blue: DAPI. Scale bar, 10 μm. The arrow indicates a lysosome with collagen. **C)** Surface expression of CD206 in macrophages differentiated with or without 5-MTP upon activation for 5 h by IL-4. Shown is the geometric mean fluorescence intensity from flow cytometry (*n* = 5 donors, paired *t*-test). **D)** TNF-α production of macrophages stimulated for 5 h with LPS and in absence or presence of 5-MTP (*n* = 5 donors, one-way ANOVA with a Dunnette’s multiple comparison test). Data points show individual donors. (For interpretation of the references to colour in this figure legend, the reader is referred to the Web version of this article.)

Experiments using FITC-labelled collagen demonstrated that macrophages differentiated with M-CSF and 100 μM 5-MTP and subsequently polarised with IL-4, showed a ~10% increased collagen uptake, which was trafficked to the lysosomes without affecting cell viability (Fig. 1A and B; Supplementary Figs. 1A–B). Moreover, 5-MTP-differentiated macrophages showed an about ~10% increased surface levels of CD206, a receptor mediating the uptake of collagen [20–22], compared to differentiation without 5-MTP, although we did not observe changes in other anti-inflammatory factor *TFGB1* (Fig. 1C; Supplementary Figs. 1C–D). 5-MTP has been shown to affect production of interleukin-6 (IL-6) and TNF-α in mouse macrophage models [17]. However, we did not observe any significant changes in production of TNF-α and IL-6 in human macrophages when 100 μM 5-MTP and LPS were added simultaneously to macrophages in culture media (Fig. 1D; Supplementary Fig. 1E). Moreover, the expression of macrophage-associated markers CD14 and CD16 was also unaltered upon differentiation in the presence of 5-MTP (Supplementary Fig. 1F). Thus, the uptake of collagen by macrophages is promoted if they differentiated in the presence of 5-MTP, but this does not affect LPS-induced production of TNF-α or IL-6 during inflammation.

### 2.2 5-MTP affects membrane fluidity of mitochondria

**Fig. 2:**
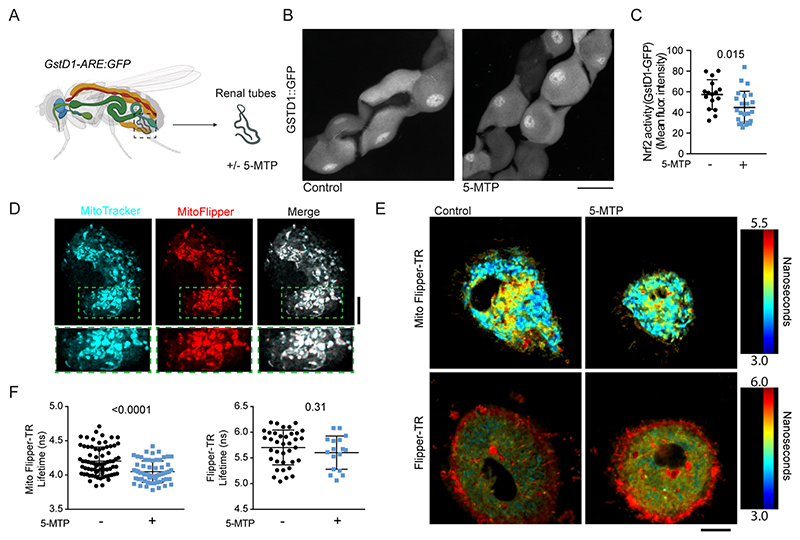
5-MTP affects the membrane fluidity of mitochondria. **A)** Schematic overview of experimental setup for *Drosophila melanogaster* renal tube extraction and analysis under the microscope. **B)** *Drosophila* renal tubules from the NRF2 activity reporter (Glutathione S-transferase D1 (GstD1):GFP, a target of NRF2-transcription) after incubation with and without 5-MTP. Scale bar, 20 μm. **C)** Quantification of GstD1:GFP levels induced by NRF2-driven transcription in the presence or absence of 5-MTP. Datapoints show the mean fluorescent intensity measured across the main segment of one single renal tubule (two-sided unpaired *t*-test). **D)** Confocal microscopy of human macrophages labelled with Mitotracker MF Green (cyan) and Mito Flipper-TR (red). **E)** FLIM images of macrophages differentiated with and without 5-MTP and labelled with Mito Flipper-TR and Flipper-TR. **F)** Average apparent fluorescence lifetimes per cell of Mito Flipper-TR and Flipper-TR. (*n* = 3 donors, two-sided unpaired *t*-test). Datapoints indicate individual cells. (For interpretation of the references to colour in this figure legend, the reader is referred to the Web version of this article.)

Next, we investigated the mechanism of how 5-MTP influences the macrophages. Previously, 5-MTP was shown to induce NRF2 signaling [15]. Therefore, we investigated the expression of the NRF2 regulated genes coding for heme oxygenase-1 (*HO-1*) and NAD(P)H quinone dehydrogenase 1 (*NQO-1*). However, we did not find any alterations of expression of these genes in 5-MTP-differentiated macrophages (Supplementary Fig. 2). We also investigated the effects of 5-MTP in an *in vivo* model for NRF2 signaling, by incubating extracted renal tubules from GstD-ARE:GFP transgenic *Drosophilla melanogaster* with 5-MTP [23]. These reporter flies express green fluorescent protein (GFP) downstream of the Glutathione S transferase D1 (GstD) promoter sequence, a target of NRF2 signaling (Fig. 2A–C). We found that 5-MTP incubation did not promote GstD expression as expected, but instead decreased its expression. Thus, we could not confirm that 5-MTP induces NRF2 signaling.

These results motivated us to search for other mechanisms by which 5-MTP influences the macrophages. Since 5-MTP has been reported to influence mitochondria [24], and as mitochondrial functions relate to macrophage functions [3,10], we speculated that 5-MTP might affect the mitochondria. 5-MTP is an amphipatic molecule expected to partion in lipid membranes and alter their physicochemical properties such as lateral membrane tension. We therefore investigated whether 5-MTP would affect the membrane fluidity of the mitochondria using the lateral membrane tension probe Mito Flipper-TR [25]. Mito Flipper-TR inserts into the membranes of mitochondria, as confirmed by live-cell imaging together with the mitochondrial probe MitoTracker MF Green (Fig. 2D). Fluorescence lifetime imaging microscopy (FLIM) showed that the fluorescence lifetime of Mito Flipper-TR was reduced on average by 100-200 ps when the macrophages were differentiated in the presence of 5-MTP (Fig. 2E) [25], indicating reduced membrane tension. This effect is specific for the mitochondrial membranes, since FLIM experiments with non-targeted Flipper-TR, which inserts in all cellular membranes [26], showed no differences in 5-MTP differentiated macrophages (Fig. 2F). Thus, 5-MTP does not signal via NRF2 in macrophages, but rather seems to affect the mitochondrial membrane. Although we cannot exclude indirect effects, it seems likely that this effect is caused by direct insertion of 5-MTP in the mitochondrial membranes given its amphipatic nature.

### 2.3 5-MTP promotes mitochondrial branching and protects against lipid oxidation

**Fig. 3:**
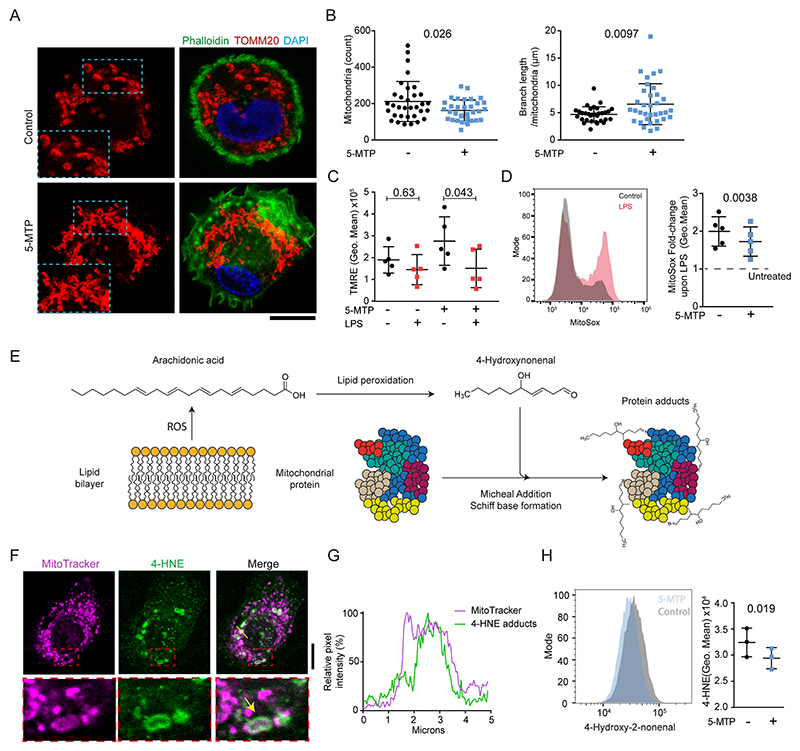
5-MTP promotes mitochondrial network formation and reduces lipid oxidation. **A)** Confocal images of macrophages differentiated with and without 5-MTP and immunostained for TOMM20 (red). Green, phalloidin. Blue, DAPI. Scale bar, 10 μm. **B)** Morphometry of mitochondria from 3-dimensional confocal imaging. Count: average number of mitochondria per cell. Branch length/mitochondria: average length of the extensions per mitochondria. (datapoints indicate individual cells pooled from 3 donors, two-sided unpaired t-tests) **C)** Flow cytometry analysis of TMRE signal of macrophages differentiated with and without 5-MTP and treated for 5 h with 100 ng/μl LPS. Datapoints indicate individual donors. Difference between control and 5-MTP untreated, paired *t*-test p = 0.1344 (*n* = 5 donors, two-way ANOVA with a Sidak’s multiple comparisons test). **D)** Flow cytometry histogram and relative normalised MitoSox staining of macrophages differentiated with and without 5-MTP and treated for 5 h with LPS (*n* = 5 donors, two-sided paired *t*-test). Data points indicate individual donors. **E)** Schematic overview of ROS-promoted lipid oxidation generating 4-HNE. 4-HNE forms adducts with proteins via Schiff base formation and Michael addition. **F)** Confocal microscopy of macrophages stained with MitoTracker (magenta) prior to fixation and immunofluorescent staining with an antibody against 4-HNE (green). Scale bar, 10 μm. **G)** Fluorescence intensity profile graphs from the cross-sections indicated in the merge image of panel F. **H)** Flow cytometry histogram and quantification of geometric mean fluorescence intensities of 4-HNE (*n* = 3 donors, two-sided paired *t*-test). Datapoints indicate individual donors. (For interpretation of the references to colour in this figure legend, the reader is referred to the Web version of this article.)

Since we found that 5-MTP decreases the tension of mitochondrial membranes, we expected that this would decrease mitochondrial fission [27], a hallmark of anti-inflammatory macrophages [10]. To address this, the cellular organization of the mitochondria was characterised using 3-dimensional confocal imaging of macrophages stained for the mitochondrial protein TOMM20 (Fig. 3A and B and Supplementary Fig. 3A). Indeed, we found less fragmented, more branched and elongated mitochondria in 5-MTP-differentiated macrophages.

To test functional differences of the mitochondria in 5-MTP differentiated macrophages, we incubated the cells for short times (<5 h) with LPS. LPS triggers depolarisation of the inner membranes of mitochondria by creating a shunt in the electron transport chain, thereby breaking the TCA cycle and promoting ROS production [28]. Since our data shows that the mitochondria are more connected upon differentiation in the presence of 5-MTP, whereas they are more fragmented without 5-MTP, we hypothesised that LPS would increase the level of overall mitochondrial membrane depolarisation in 5-MTP differentiated macrophages. Indeed, using the mitochondrial membrane potential (ΔΨm) probe tetramethylrhodamine ethyl ester (TMRE), we observed a stronger membrane depolarisation in the macrophages differentiated with 5-MTP (Fig. 3C).

Fragmented mitochondria are regarded as a stronger ROS-producing phenotype in macrophages that are activated by pathogenic stimuli [10, 29]. Because we found less fragmented mitochondria in 5-MTP-differentiated macrophages and the Mito Flipper-TR experiments indicate that 5-MTP affects the mitochondria, we decided to measure subcellular localisations of ROS formation and quantify the amount of 4-HNE protein adducts (an end-product of lipid oxidation).

We used MitoSox, a probe for ROS levels at the mitochondria. We found reduced MitoSox labeling in LPS-stimulated macrophages and Drosophila renal tubules in the presence of 5-MTP (Fig. 3D; Supplementary Fig. 3B). These results indicated a relative reduction of mitochondrial ROS in the presence of 5-MTP. Oxidation of omega-6 polyunsaturated fatty acids (*i.e*. linoleic acid or arachidonic acid) in cellular membranes by ROS results in the production of 4-HNE [30,31]. 4-HNE is a reactive aldehyde product that forms covalent adducts with proteins that can be detected by antibody labelling (Fig. 3E), and in human macrophages, 4-HNE is formed in mitochondria (Fig. 3F and G). By quantifying the levels of 4-HNE protein adducts with flow cytometry we found that the amount of these adducts was ~20% reduced in 5-MTP differentiated macrophages (Fig. 3H), indicating that 5-MTP reduces lipid oxidation of mitochondrial membranes. Since 4-HNE promotes NRF2 signaling by modification of the inhibitory protein KEAP1 [32–34], the inhibition of 4-HNE production by 5-MTP might explain the observed absence of NRF2 signaling in human macrophages and the reduction in *Drosophila* renal tubules. This indicates that 5-MTP might protect against ROS-induced damage.

NAPH oxidases residing in endosomes of the macrophage are also potent producers of ROS [35]. However, we did not observe any effect of 5-MTP on lipid oxidation at endosomes (Supplementary Figs. 3C–E). Moreover, at least in dendritic cells, mitochondrial ROS contribute to the breakdown of ingested antigens [36]. To test if 5-MTP would affect antigen processing, we used double-quenched ovalbumin (DQ-OVA), a model antigen that is degraded in lysosomes as demonstrated by experiments with the v-ATPase inhibitor Bafilomycin A (Supplementary Fig. 3F). Indeed, when macrophages were differentiated with 5-MTP, the proteolysis of ingested DQ-OVA in vacuoles was reduced significantly while OVA uptake was not affected (Supplementary Figs. 3G–H). These results are in line with our conclusion that 5-MTP reduced ROS production specifically at mitochondria. Thus, 5-MTP results in more branched mitochondria that produce less ROS.

### 2.4 5-MTP reduces lipid accumulation and fatty acid uptake

**Fig. 4:**
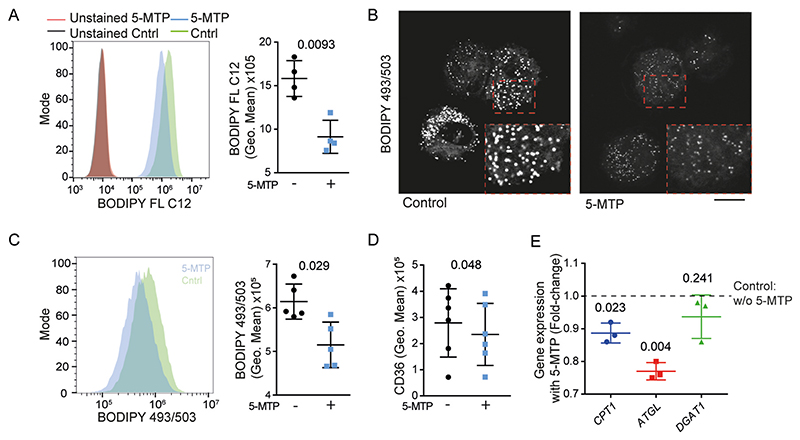
5-MTP shifts macrophages from lipid to glucose metabolism. **A)** Flow cytometry histograms and quantification of geometric mean fluorescence intensities of macrophages differentiated with or without 5-MTP. Lipid uptake was measured with BODIPY FL C12 (data points: individual donors; *n* = 4 donors, two-sided paired *t*-test). **B)** Live-cell confocal microscopy of macrophages with lipid droplets visualized by BODIPY 493/503. Scale bar, 10 μm. **C)** Flow cytometry histograms and quantification of BODIPY 493/503-staining for macrophages differentiated with or without 5-MTP (*n* = 5 donors, two-sided paired *t*-test). **D)** Flow cytometry of CD36 surface staining of macrophages differentiated with or without 5-MTP (*n* = 6 donors, two-sided paired *t*-test). **E)** RT-qPCR of *CPT1*, *ATGL*, and *DGAT1* of 5-MTP differentiated macrophages normalised to macrophages without 5-MTP (control indicated as a dashed-line) (*n* = 3 donors, two-sided paired *t*-test).

Lipids are metabolized in mitochondria. Hence, we determined whether the uptake of lipids was altered in 5-MTP-differentiated macrophages. Indeed, flow cytometry showed a ~2-fold reduction of uptake of the fluorescent lipid analogue BODIPY FL C12 by the 5-MTP-differentiated macrophages (Fig. 4A). Moreover, flow cytometry showed that the 5-MTP-differentiated macrophages contained ~30% less lipid droplets as visualized with the probe BODIPY493/503 (Fig. 4B and C).

We then investigated whether this reduced lipid uptake was the result of decreased expression of proteins involved in fatty acid metabolism. However, although surface levels of the lipid transporter CD36 [37] were significantly reduced in 5-MTP differentiated macrophages, this reduction was on average only ~15% (Fig. 4D). Similarly, the expression of the genes coding for adipose triglyceride lipase (*ATGL*) and carnitine palmitoyl transferase I (*CPT1*) [38], involved in lipid metabolism and storage (Supplementary Fig. 4A), were only 10–25% lower in 5-MTP differentiated macrophages (Fig. 4E). Moreover, expression of the genes coding for acetyl-CoA carboxylase 1 (*ACACA*) and ATP citrate synthase (*ACLY*), the rate limiting enzymes in fatty acid synthesis, were not affected by 5-MTP (Supplementary Fig. 4B). Expression of diacylglycerol *O*-acyltransferase 1 (*DGAT1*), involved in the biosynthesis of lipid droplets, was also unaltered. These changes in expression of key components of lipid metabolism are relatively minor, and likely insufficient to explain the ~2-fold reduction in lipid uptake. Therefore, we tested whether 5-MTP would affect lipid uptake irrespective of protein expression.

### 2.5 5-MTP inhibits acyl-carnitine uptake in mitochondria

**Fig. 5:**
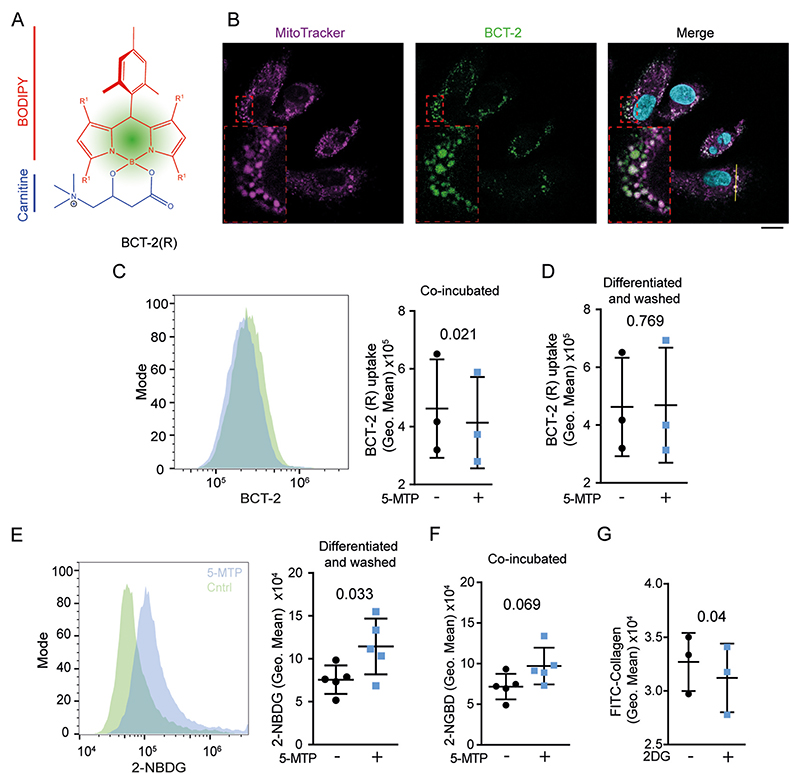
5-MTP inhibits acyl-carnitine uptake by mitochondria and promotes glucose uptake by macrophages. **A)** Schematic overview of acyl-carnitine biomimetic probe BCT-2. **B)** Confocal microscopy image of BTC-2 probe (green) co-stained with MitoTracker CMXROS red (magenta). DAPI: cyan. Scale bar, 10 μm. **C)** Flow cytometry data of macrophages treated with the BCT-2 probe in the presence of 5-MTP. Data points indicate individual donors (*n* = 3 donors, two-sided paired *t*-test). **D)** Flow cytometry data of BCT-2 uptake by macrophages differentiated in the presence of 5-MTP and subsequently washed with PBS without 5-MTP (*n* = 3, two-sided paired *t*-test). **E)** Flow cytometry of cells differentiated in presence or absence of 5-MTP and pulsed with fluorescent glucose analogue 2-NBDG (*n* = 5 donors, two-sided paired *t*-test). **F)** 2-NBDG uptake by macrophages in the presence of 5-MTP. (*n* = 5 donors, two-sided paired *t*-test). **G)** Uptake of collagen-FITC by macrophages treated with 2-deoxy glucose (2DG). (*n* = 3 donors, paired *t*-test). Data points indicate individual donors. (For interpretation of the references to colour in this figure legend, the reader is referred to the Web version of this article.)

The lipid components that are most affected in serum of fibrotic patients aside from 5-MTP are acyl-carnitines [15,16]. The rate limiting step for lipid metabolism is the import of acyl-carnitines into the mitochondria [39,40]. Using the recently developed acyl-carnitine biomimetic BCT-2 probe [41], we investigated if 5-MTP hampers the uptake of acyl carnitines into the mitochondria (Fig. 5A and B and Supplementary Fig. 5A). Indeed, the translocation of BCT-2 into mitochondria was ~30% reduced in the presence of 5-MTP (Fig. 5C). As a control experiment, we confirmed that mitochondrial import of the ‘natural’ (*R*)-enantiomer of the BCT-2 probe was more efficient than the ‘non-natural’ (*S*)-enantiomer, as previously reported [41] (Supplementary Fig. 5B). Importantly, we did not detect differences in mitochondrial uptake of the (*R*)-BCT-2 probe when the 5-MTP differentiated macrophages were washed and resuspended in media without 5-MTP (Fig. 5D). This indicates that 5-MTP only suppresses acyl-carnitine import into mitochondria when it is present, arguing against a mechanism based on altered protein expression.

We also investigated cellular uptake of glucose using the fluorescent glucose analogue 2-(N-(7-nitrobenz-2-oxa-1,3-diazol-4-yl)amino)-2-deoxyglucose (2-NBDG). Uptake of 2-NBDG was increased ~2-fold in 5-MTP differentiated macrophages (Fig. 5E). A trend (*P* = 0.0695) to increased glucose uptake was also observed in macrophages differentiated without 5-MTP, when the 5-MTP was only present during the 15 min of the glucose uptake experiments (Fig. 5F). Importantly, increased glucose uptake related to the enhanced collagen uptake in 5-MTP-differentiated macrophages, as we could inhibit collagen uptake by blocking glucose uptake with the competitive inhibitor 2-deoxy-D-glucose (Fig. 5G). Thus, 5-MTP reduces the import of acyl-carnitines into mitochondria and, likely as a compensatory mechanism, promotes glucose uptake and this in turn promotes efficient uptake of FITC-labelled collagen.

### 2.6 Pro-inflammatory stimuli promote degradation of 5-MTP by IDO-1

**Fig. 6:**
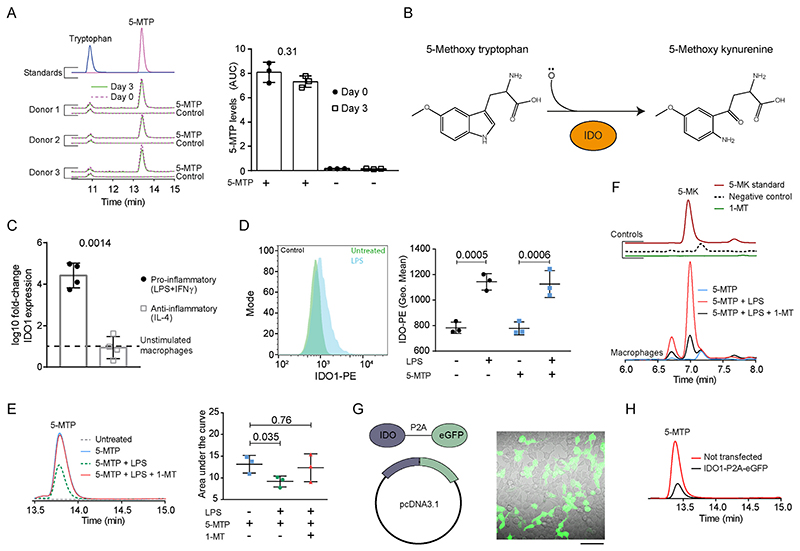
Macrophages require pro-inflammatory stimuli to degrade 5-MTP via IDO. **A)** HPLC chromatographs and quantification (area under the curve; AUC) of 5-MTP in medium cultured with macrophages with or without 5-MTP and the same sample three days later (*n* = 3 donors, one-way ANOVA with a Dunnette’s multiple comparison test). **B)** Schematic overview of 5-MTP breakdown into 5-methoxy kynurenine (5 MK) by IDO. **C)** Expression change of *IDO1* in macrophages stimulated with LPS (100 ng/ml) and IFN-γ (20 μg/ml) or IL-4 (60 μg/ml) for 24 h compared to untreated macrophages (n = 4, two-sided paired *t*-test). **D)** Flow cytometry histogram and quantification of geometric mean fluorescence intensity of IDO-1 in macrophages. Macrophages were differentiated in the presence and absence of 5-MTP and subsequently treated for 5 h with 100 ng/ml LPS (*n* = 3 donors, two-way ANOVA with a Sidak’s multiple comparisons test). **E)** HPLC chromatographs and quantification of medium from macrophages cultured with 5-MTP, activated with LPS, and in the presence of the IDO inhibitor 1 MT (*n* = 3 donors, one-way ANOVA with a Dunnette’s multiple comparison test). **F)** HPLC chromatograph showing 5 MK production in medium with 5-MTP by macrophages. **G)** Plasmid coding for IDO-1 and eGFP with a proteinase 2A linker (P2A). Representative microscopy images of IDO-1-P2A-GFP expressing constructs in human embryonic kidney (HEK) cells. Scale bars, 50 μm. **H)** HPLC chromatographs showing 5-MTP levels in culture medium by HEK cells expressing IDO-1.

Finally, we investigated why 5-MTP does not seem to affect the pro-inflammatory function of macrophages, such as LPS-induced production of TNF-α or IL-6. First, we assessed whether macrophages metabolized 5-MTP by HPLC. In absence pro-inflammatory stimuli, we found that levels of 5-MTP did not decrease during 3 days of differentiation, indicating that it was not degraded by non-inflammatory macrophages (Fig. 6A). As 5-MTP contains an indole-amine structure, we hypothesised that indole-amine oxygenase-1 (IDO-1) [42] would be able to degrade it (Fig. 6B). This enzyme converts tryptophan to kynurenine and can also convert other structurally related indole-derivatives [43]. Notably, 5-MTP levels are reduced in sepsis patients compared to healthy controls, and co-injection of 5-MTP with LPS in mice reduces the amount of kynurenines formed [17]. However, the direct role of IDO in 5-MTP degradation has not been investigated. We found that IDO1 is upregulated at both the mRNA (Fig. 6C) and protein levels upon LPS stimulation (Fig. 6D; Gating as in Supplementary Fig. 1B, 5-MTP histogram in Supplementary Fig. 6A). Moreover, co-incubation of 5-MTP overnight with LPS resulted in a ~45% decrease of the levels of 5-MTP (Fig. 6E), demonstrating that 5-MTP is degraded by LPS-activated macrophages. The increased expression of IDO1 was responsible for the degradation of 5-MTP, since treatment of the LPS-stimulated macrophages with IDO-inhibitor 1-methyl tryptophan (1-MT) blocked the degradation of 5-MTP. Moreover, we found that LPS-stimulated macrophages, but not non-activated macrophages, produce 5-methoxy kynurenine, the predicted product of IDO-mediated degradation of 5-MTP (Fig. 6F). This production of 5-methoxy kynurenine production could also be blocked by 1-MT.

To further confirm that IDO-1 degrades 5-MTP, we transiently expressed IDO-1 in human embryonic kidney (HEK) cells, which do not express this protein endogenously, and incubated these cells with 5-MTP (Fig. 6G). HPLC experiments revealed that IDO-1 can degrade 5-MTP (Fig. 6H), but it does not degrade without IDO-1 transfection and is stable in cell culture media (Supplementary Fig. 6B). We obtained similar results for IDO-2, a functional and structural homolog of IDO-1 (Supplementary Figs. 6C–D). Thus, human macrophages need to be activated with pro-inflammatory stimuli before they can degrade 5-MTP by upregulation of IDO. Since 5-MTP degradation will diminish its cellular effects, this likely explains why we did not detect effects of 5-MTP on LPS-induced TNF-α or IL-6 production in macrophages.

## 3 Discussion

The removal of excess collagen by macrophages is important for the resolution of inflammation, wound healing, and embryonic development [44,45]. In this study, we found that 5-MTP promotes the uptake of collagen by altering the immunometabolism of macrophages. Our data shows that 5-MTP has a unique mechanism of action, because it affects the structure and function of mitochondria: 5-MTP is not metabolized by non-activated macrophages, but affects their mitochondria and alters the physicochemical properties of the mitochondrial membranes. Although we cannot exclude that this effect is indirect, it likely involves direct interactions of 5-MTP with the mitochondrial membranes as 5-MTP is an amphiphilic molecule and the effect is only observed in the presence of 5-MTP.

In the presence of 5-MTP, more branched and connected mitochondria are formed, and mitochondrial ROS-production and lipid oxidation are reduced. As we show that 5-MTP reduces expression of CPT1A, the rate limiting enzyme of fatty acid oxidation, one possible mechanism for this metabolic reprogramming could be the increased production of malonyl-CoA, which inhibits CTP1A [46]. Moreover, we show that lipid import in the mitochondria is reduced by 5-MTP, and the macrophages shift to a more glucose-oriented metabolism. Supporting this reduction in lipid metabolism by 5-MTP, are findings from a metabolomics study that both 5-MTP and tiglyl-carnitines are decreased in chronic kidney disease patients [15]. Our data now suggest that these correlations might be functionally related to disease progression, as the differentiation of macrophages in the presence of 5-MTP improved the uptake of collagen. Moreover, our data suggest that this function is related to the metabolic shift, possibly because the trafficking and recycling of collagen receptors to the surface depends on glycosylation [47].

Although it is not known if and how 5-MTP affects the glycolytic flux and oxidative phosphorylation, the elevated glucose uptake and reduced lipid uptake in 5-MTP differentiated macrophages seems to contradict the canonical shift to oxidative phosphorylation in murine anti-inflammatory macrophages [3]. In inflammatory macrophages, the increased glycolysis and pentose phosphate pathway support NADPH production for ROS-production by NADPH oxidases [48], while anti-inflammatory macrophages rely more on oxidative phosphorylation and fatty acid oxidation for an efficient energy household [49]. Likely, the immunometabolism of anti-inflammatory macrophages depends on their functions in either promotion of ECM production of removal of excess ECM. However, in order to gain a full understanding of the effects of 5-MTP on the metabolic reprogramming, measurements of oxygen consumption rates and extracellular acidification rates with for example Seahorse would be useful.

Our data show that 5-MTP does not affect pro-inflammatory macrophages, because they express IDO-1 which degrades 5-MTP, while anti-inflammatory macrophages do not. This might explain the observation that IDO knockout mice have lower fibrosis symptoms after acute kidney injury [50]. Given that IDO also degrades structurally related indoles [43], and that some indoles even influence IDO activity by allosteric modification [51], it would be worthwhile to study the immunomodulatory effects of those IDO substrates and modulators in anti-inflammatory macrophages. For example, melatonin is anti-inflammatory by inhibition of COX-2 [52], but this has only been investigated in high concentrations on pro-inflammatory macrophages which express IDO. Since we show that non-inflammatory macrophages hardly express IDO compared to pro inflammatory conditions, these cytoprotective indoles might be expected to have stronger effects in this phenotypic state. Finally, the physiological relevance of our finding that 5-MTP promotes a more fibro-protective phenotype of anti-inflammatory macrophages needs to be determined with *in vivo* studies. In any case, our findings strengthen the emerging concept that 5-MTP could be a target for diseases associated with excessive collagen deposition [19].

## 4 Methods and materials

5-MTP (Sigma, M4001) was dissolved in PBS with 0.925% (w/v) HCl. 5-Methoxy kynurenine was synthesised at Chemveda Life Sciences and dissolved as 5-MTP.

## 5 Culture of human macrophages

CD14^+^ monocytes were isolated from human buffy coats (Sanquin) using MACS (Miltenyi) and 5 × 10 [6] cells were cultured in Ultra-low adherent 6-well plates (Corning) in 2 ml RPMI containing 10% FBS (Hyclone), glutamine (Lonza), antibiotics (Gibco), 5-MTP, and M-CSF (RnD 216-MC, 100 ng/ml) at 37 °C and 5% CO_2_ for seven days. On day 4, macrophages were supplemented with 1 ml medium containing 50 ng/ml M-CSF. After differentiation, the cells were washed once with room temperature PBS (Gibco) followed by the addition of cold (4 °C) PBS and incubated at 4 °C for 30 min to collect the macrophages. LPS (OB111 *E. coli*; Sigma) was used at 100 ng/ml for 5 or 24 h.

Approval to conduct experiments with human blood samples was obtained from the blood bank and all experiments were conducted according to national and institutional guidelines. Informed consent was obtained from all blood donors by the Dutch blood bank. Samples were anonymized and none of the investigators could ascertain the identity of the blood donors.

## 6 Confocal microscopy

Macrophages were seeded on glass coverslips and fixed using 4% paraformaldehyde (PFA, 15 min at 4 °C) followed by four PBS washes. Cells were blocked and permeabilised for 30 min at 4 °C with CLSM buffer (PBS +20 mM glycine +3% BSA) and 0.1% saponin followed by an overnight staining with the primary antibody (TOMM20, Abcam ab56783) 1:200 diluted in CLSM with saponin at 4 °C. The next day cells were washed twice with PBS +0.1% saponin and incubated for 30 min at room temperature in CLSM +0.1% saponin with secondary antibody donkey-anti-mouse IgG (H&L) labelled with Alexa 647 and Alexa Fluor 488-labelled phalloidin (Thermo Fisher, 10125092). After washing the cells with PBS +0.1% saponin, the coverslips were mounted on glass slides in 67% glycerol containing 1 mM Trolox and 0.33 μg/ml DAPI. Samples were imaged using an LSM800 Zeiss microscope with a 63× oil lens. Z-stacks were recorded from whole cells at 200 nm steps analysed on Fiji using the ‘Mitochondria analyser’ plug-in from A. Chaundhry et al. (2020) [53].

To visualise 4-HNE with the mitochondria, macrophages were seeded on glass coverslips after differentiation, treated with MitoTracker Red CMXRos (200 nM, 15 min, 37 °C) and fixed using 4% PFA (15 min at 4 °C) followed by four PBS washes. Cells were blocked and per- meabilised for 30 min at 4 °C with CLSM (PBS + 20 mM Glycine + 3% BSA) and 0.1% saponin followed by an overnight stain with the primary antibody against 4-Hydroxynonenal (Invitrogen, MA5-27570) in CLSM with saponin at 4 °C. The next day, the cells were washed twice with PBS +0.1% saponin and incubated for 30 min in CLSM +0.1% saponin with the secondary antibody donkey-anti-mouse IgG (H&L) Alexa fluor 488 (Thermo Scientific, 10544773). After washing the cells with PBS +0.1% saponin, the coverslips were mounted on glass slides in 67% glycerol containing 1 mM Trolox and 0.33 μg/ml DAPI. Samples were imaged using an LSM800 Zeiss microscope with a 63 ×(N/A 1.4) oil lens.

## 7 FRET/FLIM of Flipper-TR and Mito Flipper-RT

FRET-probes Mito Flipper-TR and Flipper-TR were incubated according to the company protocol on Cellview cell culture dishes (Greiner Bio-one). After incubation, the cells were washed with warm RPMI without phenol-red (containing glutamine). FLIM images were collected with a MicroTime 200 microscope (PicoQuant) equipped with an Olympus (100×/1,4) oil immersion objective. Images were acquired using the SymPhoTime 64 software (PicoQuant). Data analysis of the FLIM images was performed using the open-source FLIMfit software (Warren et al., 2013).

### 7.1 RT-qPCR

RNA was isolated using the Quick-RNA miniprep kit (R1055A) according to company protocol. After RNA-isolation cDNA synthesis was conducted with random hexamer primers (Roche, 11034731001) with the M-MLV Reverse Transcriptase kit (ThermoFisher, 28025013). cDNA was diluted in Ultra-pure water (Gibco) to 0.66 ng/μl for RT-qPCR with Power up SYBR Green Mastermix (Thermo Scientific, A25741) using the following primers for each target gene: *IDO1* (FWD: 5’AGCCCCTGACTTATGAGAACA 3’ RV: 5’ AGCTATTTCCAACAGCGCCT 3’), *SNRPD3* (FWD: 5’ GGAAGCTCATTGAAGCAGAGGAC 3’ RV: 5’ CAGAAAGCGGATTTTGCTGCCAC 3’), *TGFB1* (FWD 5’ GCAAGTGGACATCAACGGG 3’ RV: 5’ TCCGTGGAGCTGAAGCAATA 3’), *ATGL* (FWD: 5’ CCCACTTCAACTCCAAGGACGA 3’ RV: 5’ GCAGGTTGTCTGAAATGCCACC 3’), *CPT1A* (FWD: 5’ GATCCTGGACAATACCTCGGAG 3’ RV: 5’ CTCCACAGCATCAAGAGACTGC 3’), *DGAT1* (FWD: 5’ GCTTCAGCAACTACCGTGGCAT 3’ RV: 5’ CCTTCAGGAACAGAGAAACCACC 3’), *HO-1* (FWD: 5’ CCAGGCAGAGAATGCTGAGTTC 3’ RV: 5’ AAGACTGGGCTCTCCTTGTTGC 3’), *NQO1* (FWD: 5’ CCTGCCATTCTGAAAGGCTGGT 3’ RV: 5’ GTGGTGATGGAAAGCACTGCCT 3’), *ACACA* (FWD: 5’ CCAGCCACTAAGCTTGGTTCCA 3’ RV: 5’ GTAGGAGCTTGTCCTTCACCTC 3’) and *ACLY* (FWD: 5’ GCTCTGCCTATGACAGCACCAT 3’ RV: 5’ GTCCGATGATGGTCACTCCCTT 3’) with a BioRad CFX96 qPCR System.

## 8 ELISA

M-CSF-differentiated macrophages were seeded at 30,000 cells per well in a flat-bottom 96-well plate and stimulated with 5-MTP (100 μM) and LPS (100 ng/ml). Media was taken from samples after 5 h and stored at −20 °C. TNF-α ELISA was performed according to company protocols (Fisher Scientific, 15561127).

## 9 Flow cytometry

For flow cytometry, differentiated macrophages were seeded at 100,000 cell/well in a 96-well ultra-low adherence plate (Corning) and treated with probes (see table below) in the CO_2_ incubator (5%) at 37 °C. After treatment, cells were collected and resuspended in a V-bottom 96-well plate. Live cell flow cytometry (CytoFlex S, Beckman Coulter) was conducted in warm RPMI without phenol red.

For internal antibody staining for IDO1 and 4-HNE, the cells were first stained with e780 fixable live/dead staining (1:1,000, 15 min at 4 °C) prior to fixation (4% PFA, 15 min, 4 °C). Subsequently, the cells were washed multiple times with PBS. The next day, the cells were blocked and permeabilised in PBS with 2% human serum and 0.05% saponin for 30 min at 4 °C. IDO-PE (Thermo Scientific, 12-9477-42) antibody was added at 0.5 μl/10^5^ cells in 30 μl PBS with 2% human serum and 0.05% saponin and incubated for 1 h at 4 °C, followed by two washes with PBS prior to analysis. 4-HNE antibody (Thermo Fisher, MA5-27570) was diluted 1:50 and incubated for 30 min at 4 °C. Then, cells were washed with PBS +0.05% saponin and incubated with antibody donkey-anti-mouse IgG (H&L) Alexa 488 (Thermo Scientific, 10544773) for 30 min at 1:400 dilution at 4 °C. Finally, the cells were washed twice with PBS prior to analysis on a Cytoflex S flow cytometer.

**Table:** 

Probe	Concentration	Incubation time	Readout
MitoSox	1 μM	30 min	ROS levels at the mitochondria
TMRE	5 μM	30 min	Membrane potential of mitochondria
2-NBDG	50 μM	15 min	Fluorescent glucose analogue
BODIPY 493/ 503	3.5 μM	15 min	Stains lipid storage compartments
BODIPY FL C12	1 μM	15 min	Fluorescent fatty acid analogue
OVA-647	20 μg/ml	3 h	Model antigen, measures endocytosis
DQ-OVA	20 μg/ml	30 min	Model antigen, fluorescent by breakdown
BCT-2 (R)	2.5 μM	2.5 min	Fluorescent acetyl-carnitine analogue, R
BCT-2(S)	2.5 μM	2.5 min	Fluorescent acetyl-carnitine analogue, S

## 10 Collagen uptake assay

24-wells plate was coated with diluted (1:100) FITC-labeled collagen (Sigma, C4361) in PBS for 1 h at room temperature in the dark. After incubation, wells were washed with PBS two times. Next, 60,000 macrophages differentiated with or without 5-MTP were seeded into the wells and cultured for 24 h with or without 60 ng/ml IL-4 (Miltenyi Biotec, 130-093-924). The next day, the plate was washed with PBS and macrophages were detached using StemPro Accutase (Fisher Scientific, 11599686) for 10 min in the cell culture incubator followed by a e780 fixable live/dead staining (1:1,000, 15 min at 4 °C), fixation in 4% PFA, and analysis by flow cytometry. 2-Deoxy-glucose was dissolved in PBS and 20 mM was added during incubation to determine the role of sugar in collagen uptake.

## 11 ROS-sensing liposomes

Liposomes were prepared with 1 mg 1-palmitoyl-2-oleoyl-*sn*-glycero-3-phosphatidylcholine in chloroform (10 mg/ml stock, Avanti), 0.2 mg cholesterol in chloroform (50 mg/ml Avanti), and 2.5 μg BODIPY 591/581 nm C11 in DSMO (0.5 mg/ml) with an additional 1 ml of chloroform. Chloroform was removed in a rotary evaporator (40 °C, 400 mbar) and the lipid film was placed in a pressureized drystove at 23 °C for 2 days. Reconsitution of the lipid flim was done in PBS by adding glass beads and gently swirling the flask to agitate the lipid film. Next, reconsitituted lipids were loaded onto syringes for extrusion (Avestin, LiposoFast-Basic & Stabilizer) with 400 nm membrane filters for 21 passes at room temperature. 10 μl of liposomes was added to 90 μl RPMI containing 100,000 macrophages in a 96-well plate for 3 h with and without 100 ng/ml LPS. After incubation, the macrophages were washed and measured on the flow cytometer in the FITC and the ECD-channel. Analysis of lipid peroxidation was done by showing the ratiometeric FITC/ECD, to account for possible unequal dispersion in the liposome model of BODIPY C11.

For imaging of macrophage with the liposomes. Macrophages seeded on glass coverslips were incubated for 1.5 h with 50 μl of liposomes in 450 μl of RPMI followed by a fixation with 4% PFA and 4 washes of PBS. Samples were mounted with Trolox and DAPI for imaging on the Zeiss LSM800 with an (63×, NA 1.4) oil lens at 23 °C.

## 12 HPLC

300 μl RPMI medium was aspirated from 5-MTP-supplemented (100 μM) cultures of confluent macrophages. For HEK cells expression IDO1 or -2, 1 μM of hemin (Sigma, 51,280-1G) was added to the medium. The medium was mixed with 1 mL methanol at −20 °C and stored at −20C until further use. The methanol fraction was evaporated using a Savant speed-vacuum dryer (SPD131, Fisher Scientific, Landsmeer, The Netherlands) at 60 °C for 2 h. The samples were reconstituted to 300 μl with 0.7% perchloric acid. Samples centrifugated at 15,000×*g*, followed by filtration of the supernatants using 0.2 μM RC membrane (Phenomenex, Utrecht, The Netherlands). Samples were injected into the UHPLC system (Dionex UltiMate 3000 autosampler; Dionex UltiMate 3000 LPG-3400SD pump, Thermo Fisher Scientific, Waltham, Massachusetts, USA) using a C18 column (Kinetex 5 μm, C18 100 Å, 250 ×4.6 mm, Phenomenex, Utrecht, the Netherlands) at 35 °C. A gradient of water/ methanol with 0.1% formic acid (0-10 min, 95%-80% H2O; 10-20 min, 80%-5% H2O; 20-23 min, 5% H2O; 23-31 min, 95% H2O) was used with a constant flow rate of 1 mL/min. UV-detection at 260 nm was performed with an UV6000LP Detector (Dionex Ultimate 3000 variable wavelength detector, Thermo Fisher Scientific, Waltham, Massachusetts, USA). Data recording was performed using Chromeleon software (version 6.8 SR12).

## 13 Drosophila renal tubules

Drosophila stocks were maintained according to standard protocols [54]. Nrf2 activity was measured *in vivo* using the GstD-ARE:GFP transgenic reporter [55]. For imaging of adult Drosophila renal tubules, intact living animals were rapidly dissected in fresh Schneider’s medium (Sigma, S0146) using forceps and incubated in Schneider’s medium with or without 5-MTP (200 μM) at 25 °C for 1 h. Renal tubules were subsequently stained with MitoSOX (5 μM) for 5 min at 25 °C in dark conditions before several washes in Schneider’s and mounting in 15 μl Schneider’s medium for imaging. Confocal imaging was performed on a Leica TCS SP8 confocal microscope. Image analysis was performed using ImageJ (NIH) software.

## 14 Statistical Analysis

Statistical analysis was done using Graphpad software. Two-sided paired Student’s t-tests were applied to determine significance, unless sample sizes between conditions varied (*e.g*., FRET/FLIM and confocal mitochondria analysis) then a two-sided unpaired Student’s t-test was used. For multiple comparisons, two-way or one-way ANOVA was applied dependent on the experimental set-up. Significance level alpha threshold is indicated by a *P*-value below 0.05.

## Supplementary Material

Supplementary data to this article can be found online at https://doi.org/10.1016/j.freeradbiomed.2022.06.235.

Supplementary Material

## Data Availability

All raw data, including phyton scripts, have been deposited to Zenodo.
