# Supplementary material for: Mitochondrial interaction of fibrosis-protective 5-methoxy tryptophan enhances collagen uptake by macrophages
Source: Free Radic Biol Med. Author manuscript; Available in PMC 2023 Jun 23. (PMC7614693; doi:10.1016/j.freeradbiomed.2022.06.235)
Supplement: Supplementary Material [file EMS177386-supplement-Supplementary_Material.docx]

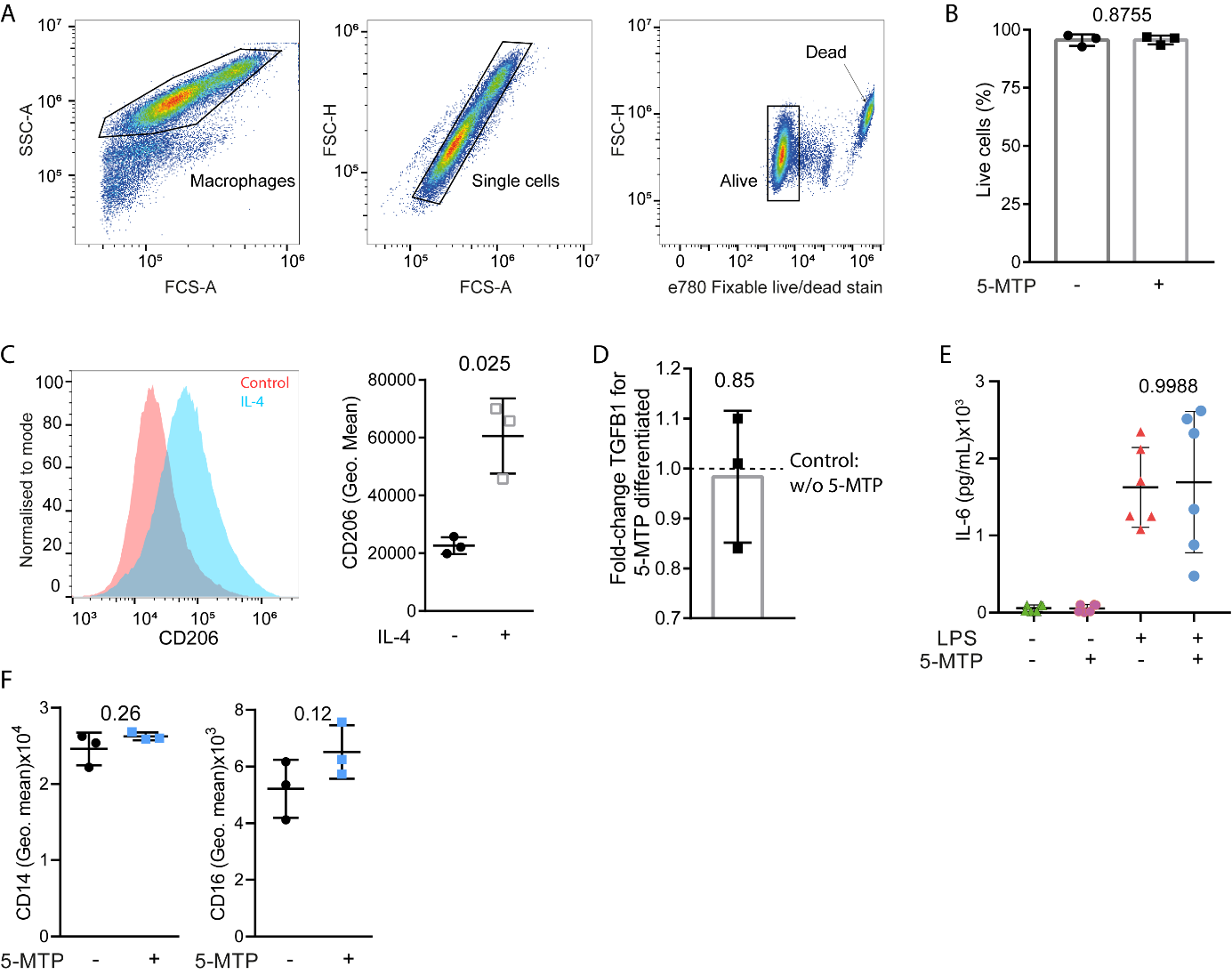


**Supplementary Figure 1. CD206 expression promoted by interleukin-4. 5-MTP does not influence IL-6 secretion.** **A)** Gating strategy of flow cytometry experiments of fixed and internally stained macrophages. In this sample, macrophages were treated with a heat-shock to show dead cells for the e780 live/dead stain. Cells were gated on side (SSC-A) and forward scatter (FSC-A), followed by a gating for single cells with FSC-area versus FSC-height and another gating for cells negative for the e780 fixable viability dye. **B)** Percentage of live (i.e., e780 negative) macrophages differentiated with and without 5-MTP determined by flow cytometry (n=3 donors, two-sided paired t-test). **C)** 24 hour incubation of IL-4 (20 ng/ml) in M-CSF differentiated macrophages (*n*=3 donors, two-sided paired t-test). **D)** RT-qPCR of *TGFB1* of 5-MTP differentiated macrophages normalised to macrophages without 5-MTP (control indicated as a dashed-line) (*n* = 3 donors, two-sided paired t-test). **E)** Interleukin-6 secretion after 24 hours of LPS stimulation in the presence of 5-MTP (*n*=5 donors, one-way ANOVA with a Dunnette’s multiple comparison test). **F)** Surface levels of macrophage markers CD14 and CD16 after differentiation with and without 5-MTP. Data points show individual donors.

**
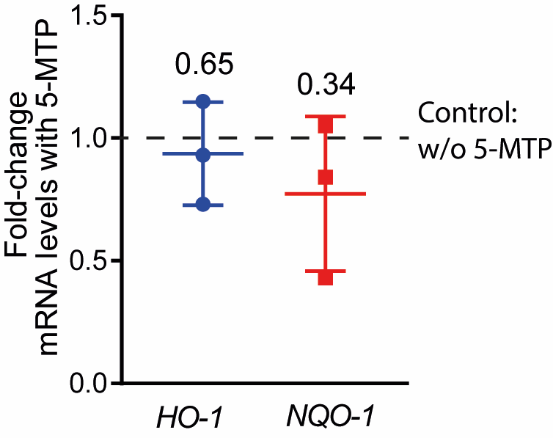
**

**Supplementary Figure 2.** qPCR for expression levels of genes downstream of NRF2 signalling in 5-MTP differentiated macrophages normalised to macrophages without 5-MTP (control indicated as a dashed-line; *n*=3 donors).


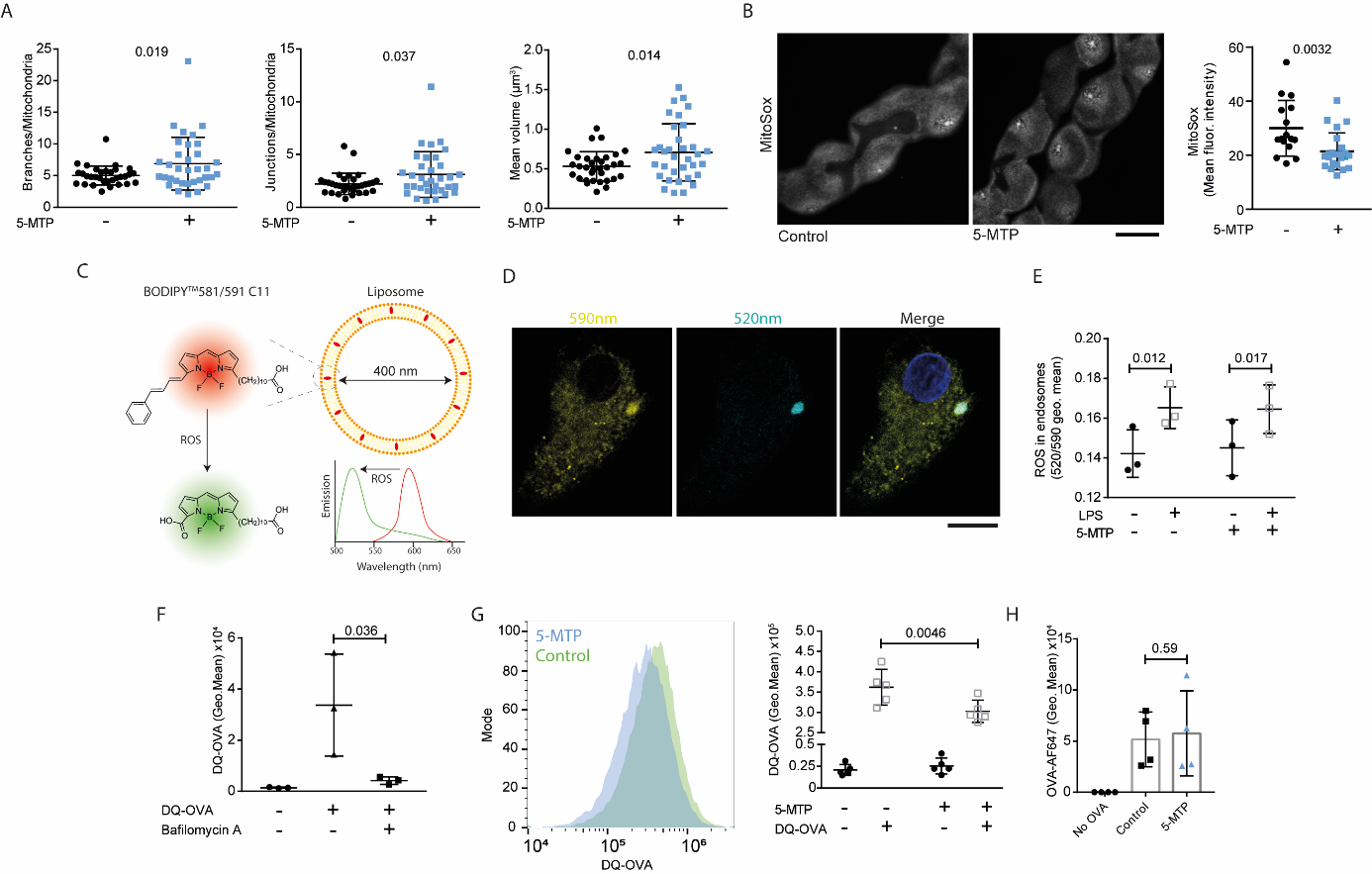


**Supplementary Figure 3. 5-MTP does not reduce endosomal ROS production, but reduces MitoSox, and reduces antigen degradation. A)** Parameters of the mitochondria analyser plugin that indicate fusion of mitochondria in macrophage differentiated in the presence of 5-MTP (see main Fig. 3A-B). Mean volume: average mean volume of individual mitochondria. Branches/mitochondria: average number of branches per mitochondria. Junctions/mitochondria: average number of joints per mitochondria. **B)** MitoSox staining in renal tubules from *Drosophila* *melanogaster* stained with MitoSox after incubation with and without 5-MTP. Scale bar, 20 µm. Graph: Relative MitoSox staining intensities in renal tubules incubated with and without 5-MTP. Datapoints indicate the mean fluorescent intensity measured across the main segment of one single renal tubule (two-sided unpaired t-test). **C)** Schematic overview of ROS-sensitive liposomes as model particles to measure ROS in endosomes. Liposomes contained the ROS-sensitive BODIPY 581/591 C11 probe. The emission of BODIPY 581/591 C11 shifts upon oxidation. **D)** Confocal microscopy of macrophages incubated with liposomes containing BODIPY 581/591 C11. The unoxidized form is in the 590 nm emission channel (yellow) and the oxidised form is in the 520 nm emission channel (cyan). The merge contains DAPI (blue). Scale bar, 10 µm. **E)** Ratio of 520 over 590 nm emission of BODIPY 581/591 C11 probe after 3 hours of uptake with and without LPS from flow cytometry. **F)** DQ-OVA processing (30 min incubation) in macrophages upon inhibition of the v-ATPase with Bafilomycin A (1 µM; n=3, one-way ANOVA with a Dunnette’s multiple comparison test) **G)** Histograms and geometric mean fluorescence intensity of flow cytometry of macrophages differentiated with and without 5-MTP and incubated with double-quenched ovalbumin (DQ-OVA) (*n*=5, two-way ANOVA with a Sidak’s multiple comparisons test). **H)** Geometric mean fluorescence intensity of flow cytometry of macrophages incubated with Alexa fluor 647-labelled ovalbumin. (*n*=4 donors, two-sided paired t-test). Data points indicate individual donors.


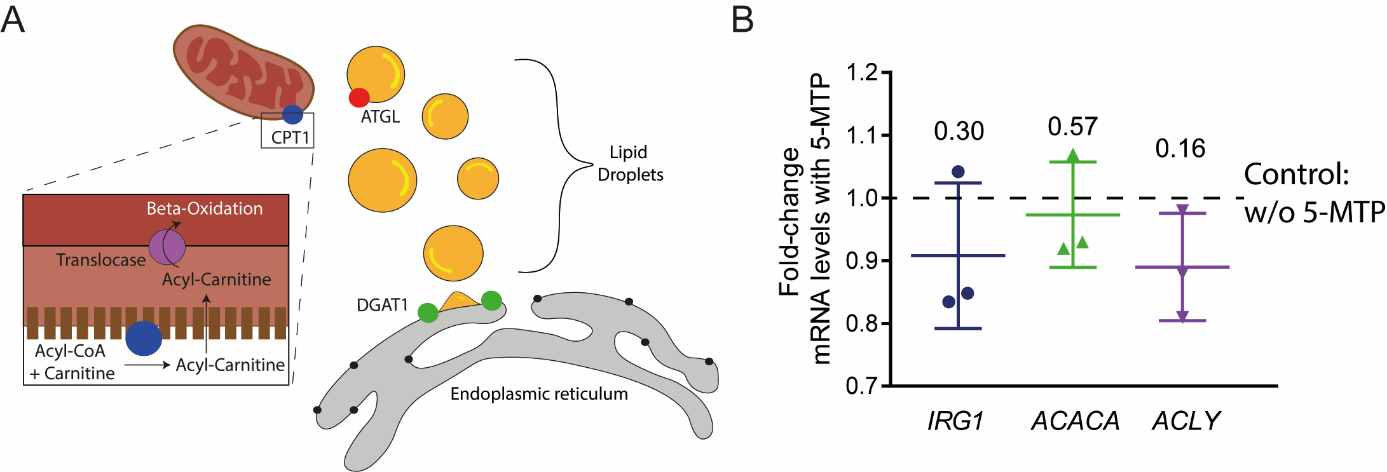


**Supplementary Figure 4. Control experiments for metabolic shift induced by 5-MTP. A)** Schematic overview of lipid droplet biosynthesis and its key enzymes for triglyceride storage and breakdown. DGAT1 plays a role in the formation of lipid droplets. Fatty acids are hydrolysed from lipid droplets by ATGL. Free fatty acids are conjugated to carnitine to allow import into the mitochondria by CPT1. **B)** qPCR for expression levels of involved in fatty acid synthesis in 5-MTP differentiated macrophages normalised to macrophages without 5-MTP (control indicated as a dashed-line; *n*=3 donors).

**
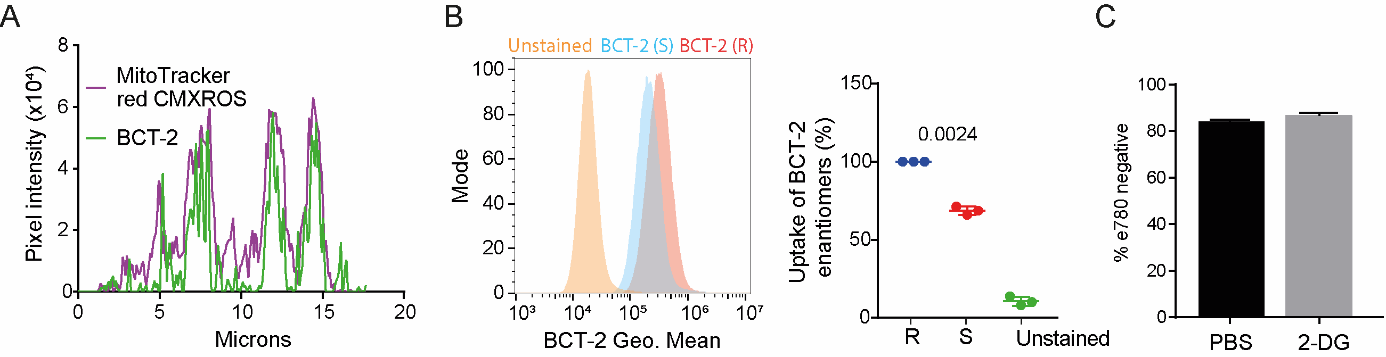
**

**Supplementary Figure 5. Control experiments for BCT-2 probe and viability of 2-DG treatment. A)** Profile plot of line drawn in microscopy image as seen in figure 5B. **B)** Left: Representative histograms of macrophages stained with BCT-2 probe ‘natural’ (*R*)- and ‘non-natural’ (*S*)-enantiomers. Right: Quantification of uptake of the BCT-2 enantiomers relative to the (*R*)-enantiomer (*n*=3, two-sided paired t-test). **C)** e780 live/dead fixable staining of the same experiment as in figure 5G, showing the percentage of living cells from the total population of single cells.


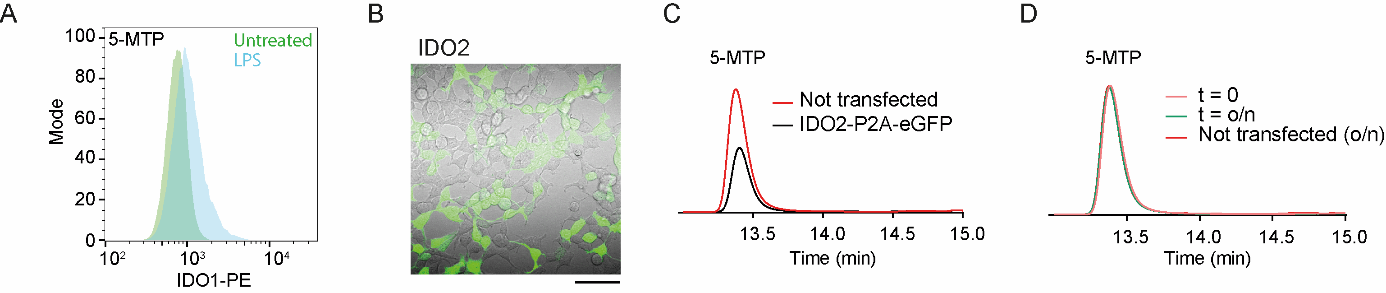


**Supplementary Figure 6. IDO2 degrades 5-MTP. A)** representative histograms of internal IDO1 staining of 5-MTP differentiated macrophages as seen in Fig. 6D. **B)** IDO2-P2A-eGFP construct expressed in HEK293 cells. Scale bars, 50 µm. **C)** HPLC histograms of IDO2-P2A-eGFP construct expressing cells superimposed over HEKs that do not express any constructs. **D)** Control conditions: HPLC histograms of HEK293 incubated with 5-MTP (Not transfected (o/n)), 5-MTP in media without any cells after addition (t=0) and after overnight incubation at 37°C (t=o/n) showing no spontaneous breakdown over time (O/N: overnight).
